# Salvage radiotherapy for patients with PSA relapse after radical prostatectomy: a single institution experience

**DOI:** 10.1186/1471-2407-8-26

**Published:** 2008-01-29

**Authors:** Laurent Quero, Pierre Mongiat-Artus, Vincent Ravery, Claude Maylin, François Desgrandchamps, Christophe Hennequin

**Affiliations:** 1 Department of Radiation Oncology, Saint Louis Hospital, 1 avenue Claude Vellefaux, 75010 Paris, France; 2 Department of Urology, Saint Louis Hospital, 1 avenue Claude Vellefaux, 75010 Paris, France; 3 Department of Urology, Bichat Hospital, 46 rue Henri-Huchard, 75018 Paris, France

## Abstract

**Background:**

To assess the efficacy of salvage radiotherapy (RT) for persistent or rising PSA after radical prostatectomy and to determine prognostic factors identifying patients who may benefit from salvage RT.

**Methods:**

Between 1990 and 2003, 59 patients underwent RT for PSA recurrence after radical prostatectomy. Patients received a median of 66 Gy to the prostate bed with 3D or 2D RT. The main end point was biochemical failure after salvage RT, defined as an increase of the serum PSA value >0.2 ng/ml confirmed by a second elevation.

**Results:**

Median follow-up was 38 months. The 3-year and 5-year bDFS rates were 56.1% and 41.2% respectively. According to multivariate analysis, only preRT PSA ≥1 ng/ml was associated with biochemical relapse.

**Conclusion:**

When delivered early, RT is an effective treatment after radical prostatectomy. Only preRT PSA ≥1 ng/ml predicted relapse.

## Background

In Europe, the estimated incidence of prostate cancer is 238,000 new cases with 85,000 deaths per year [1]. Radical prostatectomy is the most widely used treatment for localized prostate cancer. Unfortunately, local recurrences occur in up to one-third of the patients by 5 years after surgery. It is generally accepted that 30% (27–32%) of all patients by 10 years after surgery suffer biochemical relapse, defined as increasing serum prostate-specific antigen (PSA) levels >0.2 ng/ml [2,3]. PSA relapse exposes to a 34% risk of metastatic disease at 5 years. After metastatic relapse, median survival is 5 years [4].

"Salvage" radiotherapy (RT) to the prostate bed for biochemical relapse achieved biochemical control in 10–66% of the patients at 5 years [5,6]. PSA failure after prostatectomy could reflect local relapse or metastatic disease. At present, modern imaging techniques lack the sensitivity to differentiate between these two types of relapse. Identification of the best candidates for RT should be based on factors predictive for local relapse. Numbers of positive margins, low Gleason score and/or long PSA-doubling time have been proposed to select patients for RT, but they are still discussed [7].

In this study, we evaluated RT efficacy and determine prognostic factors identifying patients who may benefit from salvage RT.

## Methods

We reviewed the records of 59 patients who underwent RT between 1990 and 2003 for biochemical relapse of prostate cancer initially treated with radical prostatectomy. All patients had persistent or rising PSA >0.20 ng/ml at some time after surgery. None had imaging (bone scan and/or abdominal-pelvic computed tomography (CT) Scan) or clinical evidence of metastases at the time of the biochemical relapse.

A number of potential predictive factors were recorded: initial PSA (before surgery); age at the time of the surgery; T stage; margin status (6 sides); seminal vesicle involvement or extracapsular invasion; surgical Gleason score; perineural invasion; PSA nadir after surgery; PSA-doubling time (PSA DT) between surgery and RT calculated as follows: Ln 2 × (*t2 *- *t1*)/[Ln (PSA t2) - Ln (PSA t1)] [8]; PSA before RT (preRT PSA) and interval between surgery and RT.

RT delivered to the prostate bed a median of 66 Gy in 2.2 Gy daily fractions, four days per week, with 18 MV photon beams. Between 1990 and 1998, classical 2D RT was administered using a four-field box technique to 22 (37.3%) patients with fields of 10 cm × 10 cm shaped to protect small bowel, portions of the bladder and posterior rectal wall. The fields encompassed the prostatic/seminal vesicle bed and periprostatic tissues. Pelvic lymph nodes were not irradiated. After 1998, conformational 3D RT was adopted to define optimally the clinical target volume (CTV) and organs at risk (bladder and rectum). CTV included the prostatic/seminal bed, with a security margin to encompass subclinical disease in the periprostatic area. The planning target volume (PTV) was defined by extending the CTV 0.5 cm posteriorly and 1 cm in all other directions. No elective nodal irradiation was performed. Dose Volume Histograms were performed to decrease the dose at organs at risk. Treatment-related toxicity was graded according to the Radiation Therapy Oncology Group (RTOG) criteria [9] and the Expanded Prostate-cancer Index Composite (EPIC) score for urinary incontinence [10].

After radiation, patients were followed every 6 months by a radiation oncologist and a urologist with physical examination and PSA analysis. Imaging to exclude metastastic disease was performed at the physician's discretion, as was the prescription of hormone therapy for biochemical or clinical failure after RT. The interval between surgery and hormone therapy after RT failure was also recorded.

Biochemical failure after salvage RT was defined as an increase of the serum PSA value >0.2 ng/ml confimed by a second elevation.

Clinical failure was defined as evidence of clinical, sonographic, radiographic, or scintigraphic recurrence. The primary end point was biochemical relapse or introduction of hormone therapy before the criteria of PSA recurrence or clinical failure before biochemical relapse were met.

The other end points were overall and specific survival rates.

### Statistical analysis

Survival curves were plotted using the Kaplan-Meier method. Survival rates were calculated from the last day of RT. The date of failure was defined at the time of the biochemical failure.

Patients who biochemically had no evidence of disease (bNED) were censured at the time of last follow-up.

Univariate and multivariate analysis using a Cox proportional hazards regression analysis were conducted to identify significant predictors of biochemical outcome for several clinical and pathological factors: pre surgery PSA; Gleason score; high-grade histological differentiation (4 or 5); extra prostatic extension (capsule or seminal vesicle invasion); positive surgical margin(s); ≥3 positive surgical margins; lymphovascular invasion; short PSA DT ≤12 months; persistently high PSA after surgery (>0.2 ng/ml); preRT PSA ≥0.5, ≥1 or ≥2 ng/ml; surgery-RT interval; duration of RT; classical 2D or 3D RT.

For all analyses, the level of significance was set at 0.05.

Statistical analysis was performed using Statview software.

## Results

### Patient characteristics

Patient and tumor characteristics are reported in Table 1. Fifty-nine patients were treated within a 13-year interval. It should be noted that 11 (18.6%) patients had no lymph-node dissection at the time of radical prostatectomy and did not receive any lymph-node irradiation at the time of RT. One (2%) patient had a nodal involvement at the time of RT. Seven patients (12%) received short-term (≤6 months) hormonetherapy, with Luteinizing Hormone Releasing Hormone agonist, after surgery, at the urologist's discretion.

**Table 1:** Characteristics of the 59 men who underwent RT for PSA relapse after radical prostatectomy

Characteristic patients, n	Values
Age, median [range]	62 [46-76] years
Preoperative PSA, median [range]	16.7 [1-60] ng/ml
pTNM stage, n (%)	
pT2a	2 (3.4)
pT2b	1 (1.7)
pT2c	22 (37.3)
pT3a	20 (33.9)
pT3b	14 (23.7)
pN^-^	47 (79.7)
pN^+^	1 (1.7)
pNx	11 (18.6)
Gleason score, n (%)	
3–6	12 (20.3)
7*	30 (50.9)
3+4	13 (23.6)
4+3	13 (23.6)
8	7 (11.9)
9	10 (16.9)
Positive surgical margins†, n (%)	35/58 (60.3)
Minimal <3	19/34 (55.9)
Extensive ≥3	15/34 (44.1)
Perineural invasion, n (%)	40/46 (87)
Detectable PSA after surgery, n (%)	12 (20.3)
PSA rise after postsurgical negativity, n (%)	47 (79.7)
PSA doubling time, median	12.9 months
PSA nadir preRT, median	0.4 ng/ml
PSA level preRT, median	1.43 ng/ml
Surgery to RT interval, median	26 months

* Score composition not detailed for 4 patients.

Thirty-five patients had positive margins but no mention of margin status was made in one patient's report.

RT was delivered to 80% of the patients because of a rising PSA levels after postoperative negativity and for persistently high PSA after surgery for the remaining 20%. 3D and 2D salvage RT was performed in 63% and 37% of the patients, respectively. The median RT dose delivered was 66 Gy (59.4–70.4) in a mean of 60 days.

### Survival and bDFS

Median follow-up was 38 months after completing RT. At 3 years, the overall and specific survival rates were 93% and 100% respectively. The estimated 5-year overall survival rate was 87% and specific survival was 96%.

**Figure 1:**
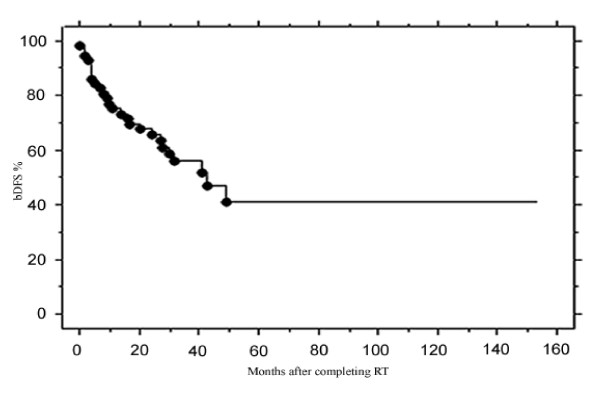
bDFS after the end of RT according to PSA >0.2 ng/ml definition.

bDFS at 3 years was 56.1%. The estimated 5-year bDFS was 41.2% (Figure 1).

### Prognostic factors for bDFS

**Figure 2:**
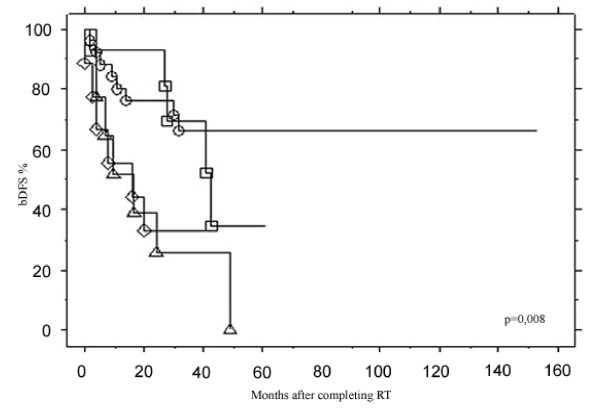
**Comparisons of bDFS after the end of RT according to different preRT PSA thresholds**. PSA < 0.5 ng/ml(○) vs. [0.5–1] ng/ml(□) vs. [1–2] ng/ml(Δ) vs. ≥2 ng/ml(◇).

**Figure 3:**
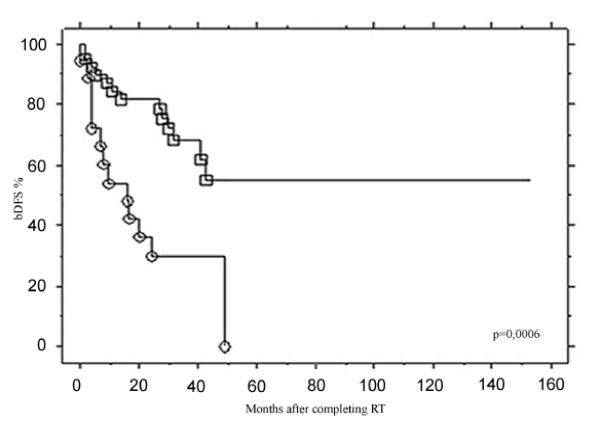
**Comparison of bDFS after the end of RT according to 1 ng/ml preRT PSA threshold**. PSA ≥1 ng/ml(○) vs. <1 ng/ml(□).

Biochemical control was analyzed using different preRT PSA thresholds (from <0.5 to ≥2 ng/ml) (Figure 2): patients with preRT PSA <0.5 or [0.5–1]ng/ml had comparable bDFS rates ≈70% while those with PSA of [1–2] or ≥2 ng/ml had ≈30% bDFS at 3 years. The bDFS rates for these different PSA thresholds were significantly different (p = 0.008). PreRT PSA of 1 ng/ml was the most significant threshold that could distinguish patients with good or bad biochemical control after RT: PSA <1 ng/ml was associated with a 3-year bDFS of 68.3% compared to 30.1% for PSA ≥1 ng/ml (p = 0.0006) (Figure 3).

PreRT PSA ≥1 (*p *= 0.001) or 2 ng/ml (p = 0.04) and not Gleason score or margin status, in particular, were associated with poor 3-year bDFS outcome (Table 2). Multivariate analysis retained only preRT PSA ≥1 ng/ml as independently predictive of biochemical failure.

**Table 2:** Prognostic factors of bDFS after salvage RT at 3 years (PSA > 0.2 ng/ml definition), univariate analysis

	3-year bDFS (%)		
Criterion	Yes	No	p-value

Presurgical PSA ≥10	58.2	48.2	0.8
Gleason score ≥8	40.3	65.2	0.2
High grade (≥4)	52.2	87.5	0.3
pT3	46.9	69.2	0.08
pT3b	41.7	60.7	0.2
Positive margin(s)	47.7	61.4	0.3
≥3 positive margins	58.3	58.8	0.7
Perineural invasion	48.3	83.3	0.8
PSA DT ≤12 months	58.8	51.9	0.8
No PSA negativity	48.6	58.2	0.2
PreRT PSA ≥2 ng/ml	33	60.9	0.04
PreRT PSA ≥1 ng/ml	30.1	68.3	0.001
Surgery-RT interval (≤12 months)	36.6	54.8	0.4
RT duration (≥60 days)	54	57.5	1.0
Classical 2D RT	70	45.7	0.2

PSA: Prostate specific antigen; DT: doubling time; RT: radiotherapy.

### Treatment tolerance

Late rectal toxicity (grade ≥2) was seen in seven patients treated with 2D RT (2 grade 3 and 2 grade 4) versus 8 with 3D RT (1 grade 3 and 0 grade 4).

Severe (grade ≥2) late urinary tract complications (stricture, hematuria) were observed in 3 patients who received 2D RT versus 1 given 3D RT, with 1 grade 3 and 0 grade 4 for each technique. Urinary incontinence (EPIC grade ≥2) developed in 2 patients given 2D RT and 4 with 3D RT.

## Discussion

We obtained a 3- and 5-year bDFS of 56.1% and 41.2% respectively, which are comparable to most of those previously reported for prostate cancer patients given RT after prostatectomy [11-31] (Table 3).

**Table 3:** Published series of salvage radiotherapy for biochemical failure after radical prostatectomy

Investigator	Year of publication	Patients (n)	Median Follow-up (months)	Freedom from Biochemical Relapse
Anscher [11]	2000	89	48	50% at 4 years
Peschel [12]	2000	39	-	27% at 3 years
Pisansky [13]	2000	166	52	46% at 5 years
Catton [14]	2001	59	44	48% at 3 years
Koppie [15]	2001	67	36	44% at 3 years
Leventis [16]	2001	49	29	43% at 3 years
Vanuytsel [17]	2001	53	36	46% at 3 years
Chawla [18]	2002	54	45	35% at 5 years
De la Taille [19]	2002	52	28	51% at 3 years
Do [20]	2002	73	87	45% at 10 years
Kalapurakal [21]	2002	41	31	57% at 5 years
Song [22]	2002	61	36	39% at 4 years
Liauw [23]	2003	51	46	56% at 3 years
Peyromaure [24]	2003	62	44	42% at 5 years
Taylor [25]	2003	44	35	66% at 5 years
Mc Donald [26]	2004	102	50	38% at 5 years
Stephenson [27]	2004	501	45	50% at 45 months
Patel [28]	2005	48	16	62.5% at 16 months
Buskirk [29]	2006	368	60	46% at 5 years
Neuhof [30]	2007	171	39	35% at 5 years
Stockdale [31]	2007	32	30	56% at 30 months
Current study	-	59	38	56% at 3 years

Poorer prognoses, in terms of bDFS after RT, were previously associated with mainly: higher preRT PSA values, high-grade disease, and seminal vesicle involvement [13-27]. Indeed, for our 59 patients, preRT PSA was associated with biochemical relapse after RT.

High preRT PSA was associated with poor biochemical control after RT regardless of the biochemical definition used. This observation is consistent with the previously reported finding that preRT PSA was the most frequently selected factor predictive of bDFS [5-31]. Those authors described poorer prognoses associated with higher PSA values before RT using thresholds ranging from 0.4 to 2 ng/ml. An ASTRO consensus panel recommended that RT be delivered before the PSA level reaches 1.5 ng/ml [32]. For our patients with a preRT PSA <1 ng/ml, the 3-year bDFS was significantly higher than for those with a PSA ≥1 ng/ml (70 vs 30% respectively). We analyzed 3-year bDFS as a function of different preRT PSA thresholds: rates declined as PSA concentrations increased from <0.5 (66.4%) to ≥2 ng/ml (only 33%). These significantly different rates (*p *= 0.008) are strong arguments supporting early treatment after biochemical relapse. We think that, in the setting of RT, the earlier the better. When biochemical failure is confirmed, and a sufficient number of factors suggestive of local relapse are present, patients should be irradiated without waiting for PSA to reach 1 or 1.5 ng/ml.

Recently, the randomized EORTC 22911 study demonstrated significant improvement for adjuvant RT vs salvage RT in terms of bDFS and clinical local control at 5 years but not for overall survival [33]. Similarly, adjuvant RT significantly increased bDFS vs observation in the ARO 96-02 and the SWOG 8794 studies [34,35]. But adjuvant RT based only on unfavorable histological prognostic features (positive surgical margins, seminal vesicle involvement or extra capsular effraction) would expose some of these patients to over treatment with the risk of incontinence and urethral stricture resulting from the accumulation of the two treatments. Indeed 40–50% of the patients with positive surgical margins would develop biochemical relapses at 5-years [36,37]. To conclude definitively, we should compare adjuvant radiotherapy vs early salvage radiotherapy: GETUG and RADICALS ongoing trials will try to answer to this question.

Gleason score and high grade prostate cancer was associated with poor biochemical outcome in previous reported studies [19-30]. However, we did not find any significant bDFS difference for patients with a low (≤6) or high (≥7) Gleason scores. Seminal vesicle involvement or margin status was also previously associated with poorer outcome.

In this study, we did not observe any difference in terms of incontinence (grade ≥2) according to EPIC score or rectal toxicity (grade ≥2) between salvage 3D and 2D RT. Urinary tract toxicities (stricture, hematuria) were also similar for the two techniques, with very low frequencies in both groups which is consistent with the MSKCC experience, in which adjuvant/salvage 3D RT was associated with 5% grade ≥2 toxicity [38].

## Conclusion

Salvage RT is an effective treatment after radical prostatectomy. bDFS 3- and 5-years after salvage RT were 56% and 41%, respectively. RT was well tolerated in terms of urinary toxicity, especially with 3D RT. PreRT PSA was the most powerful prognostic of bDFS before RT delivery and surpassed all other factors evaluated. To increase its efficacy, RT should be given earlier after biochemical relapse, ideally when preRT PSA <1 ng/ml, to obtain the best biochemical control.

## Competing interests

The author(s) declare that they have no competing interests.

## Authors' contributions

LQ wrote the manuscript, collected and interpreted the data, performed the analysis and reviewed the literature. PMA performed the surgical treatment and revised the manuscript.

VR performed the surgical treatment and revised the manuscript. CM performed the radiotherapy treatment and revised the manuscript. FD performed the surgical treatment and revised the manuscript. CH was involved in the writing of the manuscript, performed the statistical analysis and revised the manuscript. All authors read and approved the final manuscript.

## Pre-publication history

The pre-publication history for this paper can be accessed here:


